# Supplementary material for: Structure of the bacterial plant-ferredoxin receptor FusA
Source: Nat Commun. 2016 Oct 31;7:13308. doi: 10.1038/ncomms13308 (PMC5095587; doi:10.1038/ncomms13308)
Supplement: Supplementary Information — Supplementary Figures 1-13 and Supplementary Tables 1-3. [file ncomms13308-s1.pdf]

Supplementary Information

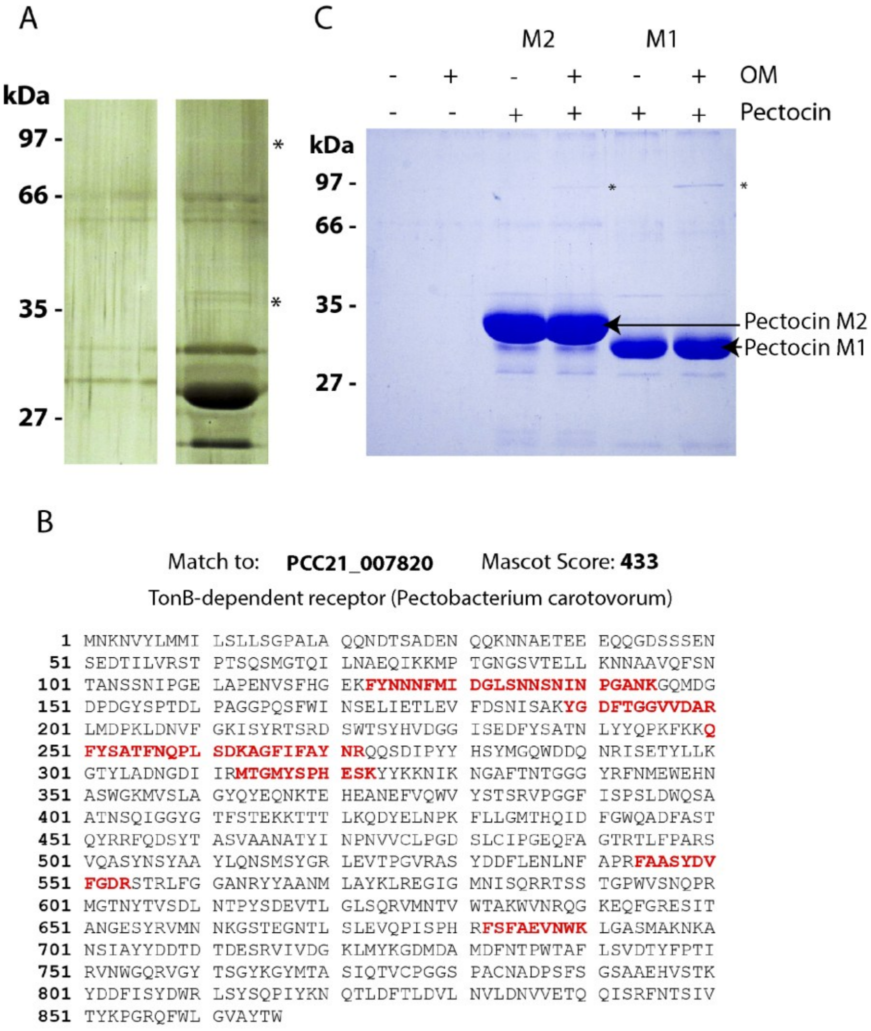

Supplementary Figure 1: Co-purification of *Pectobacterium* membranes with pectocin M1 identifies the ferredoxin receptor FusA. A) Silver stained SDS-PAGE gel showing co-elution of FusA with His-tagged pectocin M1 from nickel affinity resin. Solubilised outer-membranes from *Pectobacterium carotovorum* LMG 2386 had been passed over the resin in the absence (lane 1) and presence (lane 2) of bound pectocin M1, before washing and elution with imidazole. Stars represent proteins that co-eluted in the presence of pectocin M1; the upper band at approximately 97 kDa was identified as FusA, while the lower band was identified as the abundant outer membrane protein OmpA. B) Peptides identified by tandem MS from the approximately 97 kDa band of the gel from A), identifying the protein as a TonB dependent receptor of unknown function, designated FusA. C) Coomassie stained SDS-PAGE gel showing co-elution of FusA with pectocin M1 and M2 when recombinantly expressed in *E. coli* BL21 (DE3). (All experiments performed 2 times)

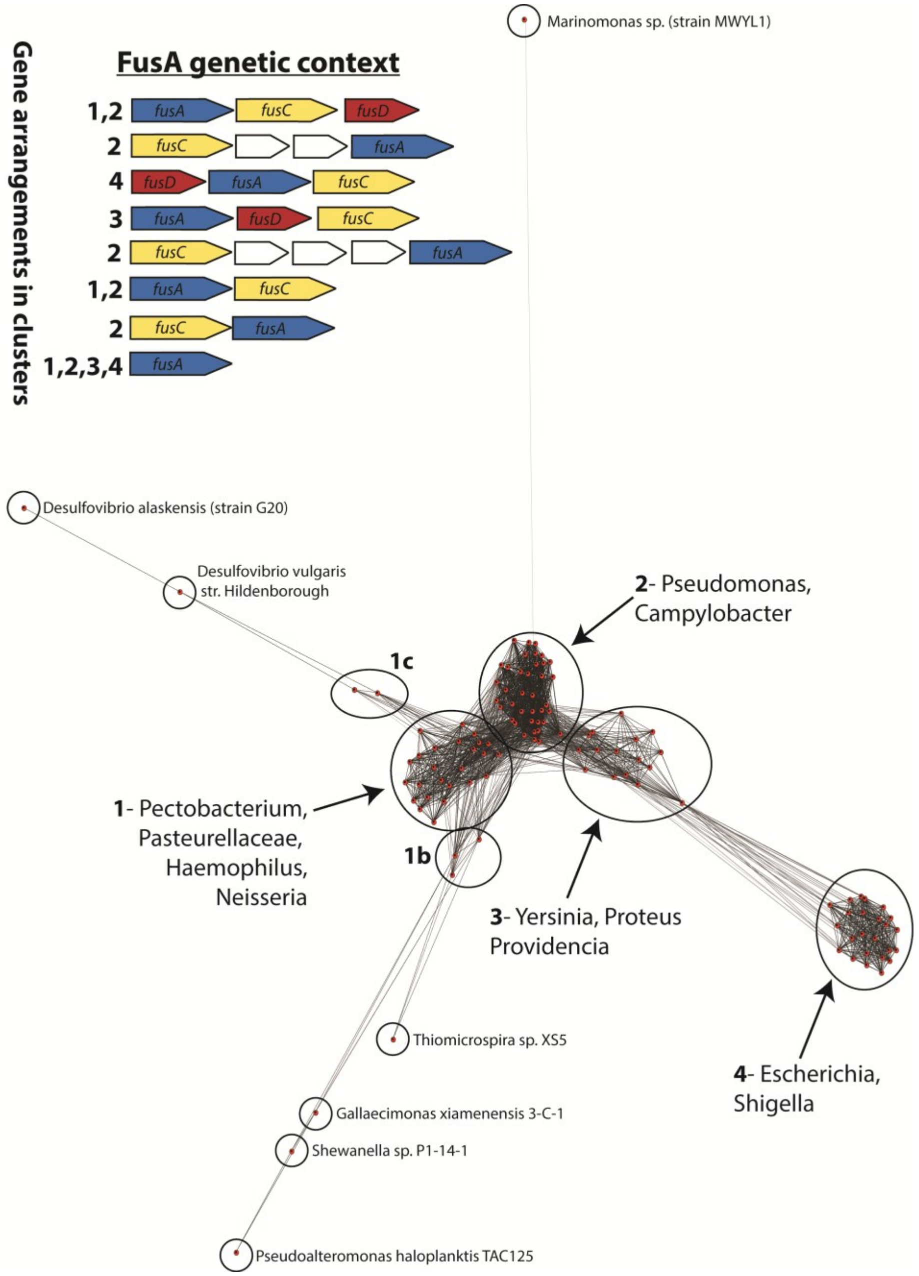

Supplementary Figure 2: Clustering of FusA homologues determined using CLANS. A) CLANS derived clustering of FusA homologues identified by HMMER search of the Uniprot rp75 sequence database. A table of the sequences utilised for this clustering analysis are listed in Supplementary Table 1. Four clusters were identified corresponding FusA homologues from different bacterial lineages. The genetic context (the putative Fus operon) of *fusA* is shown for the different clusters with *fusC* (M16 family protease) homologues present in each group, with a variation observed in the order of the Fus genes. White open reading frames are hypothetical proteins.

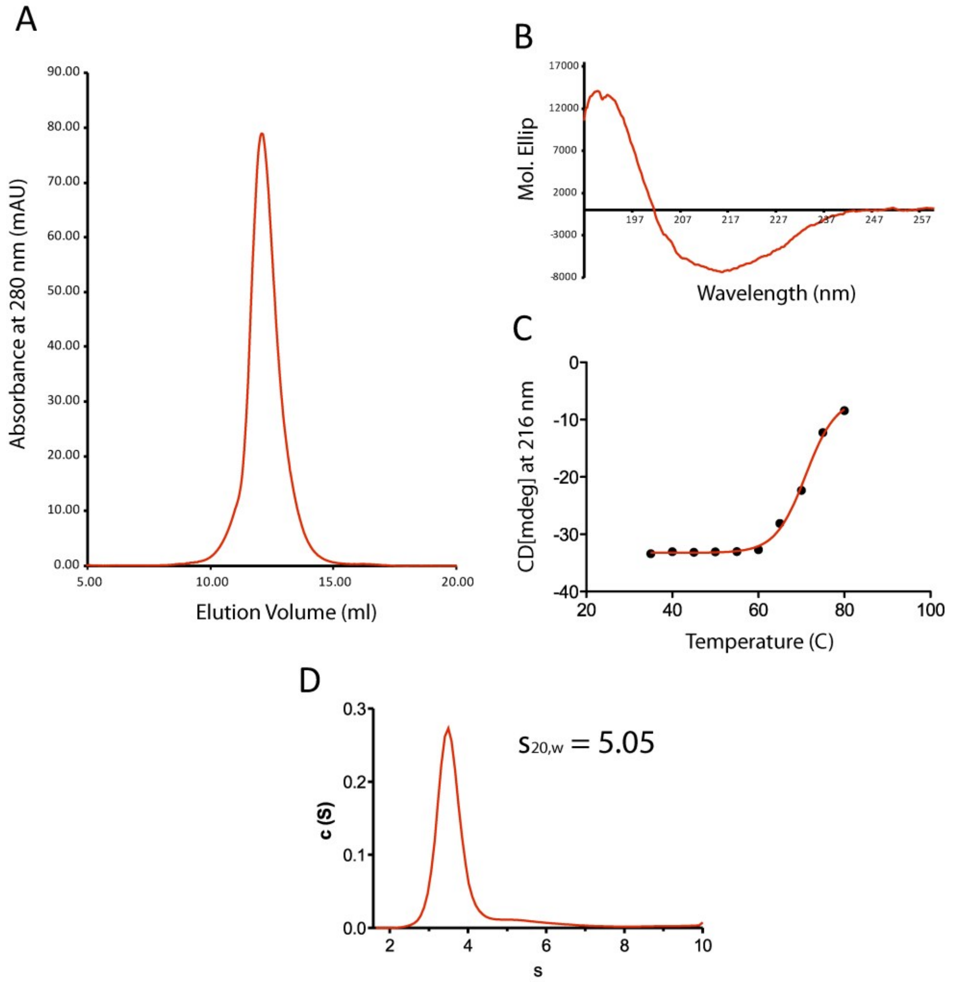

Supplementary Figure 3: Validation of FusA refolding. A) Analytical Superdex S200 gel filtration profile of purified FusA, shows a single species with Gaussian elution profile. B) Far-UV CD spectrum for purified FusA suggests a predominantly β-sheet structure. C) The melting profile with a discrete sigmoidal unfolding curve with a mid-point of 72.5°C suggests a folded, highly stable structure. D) Sedimentation velocity data for purified and refolded FusA (10 µM) shows that the protein is monodisperse and monomeric in solution, with a sedimentation coefficient (s20, w) of 5.05 S. (All experiments performed 2 times)

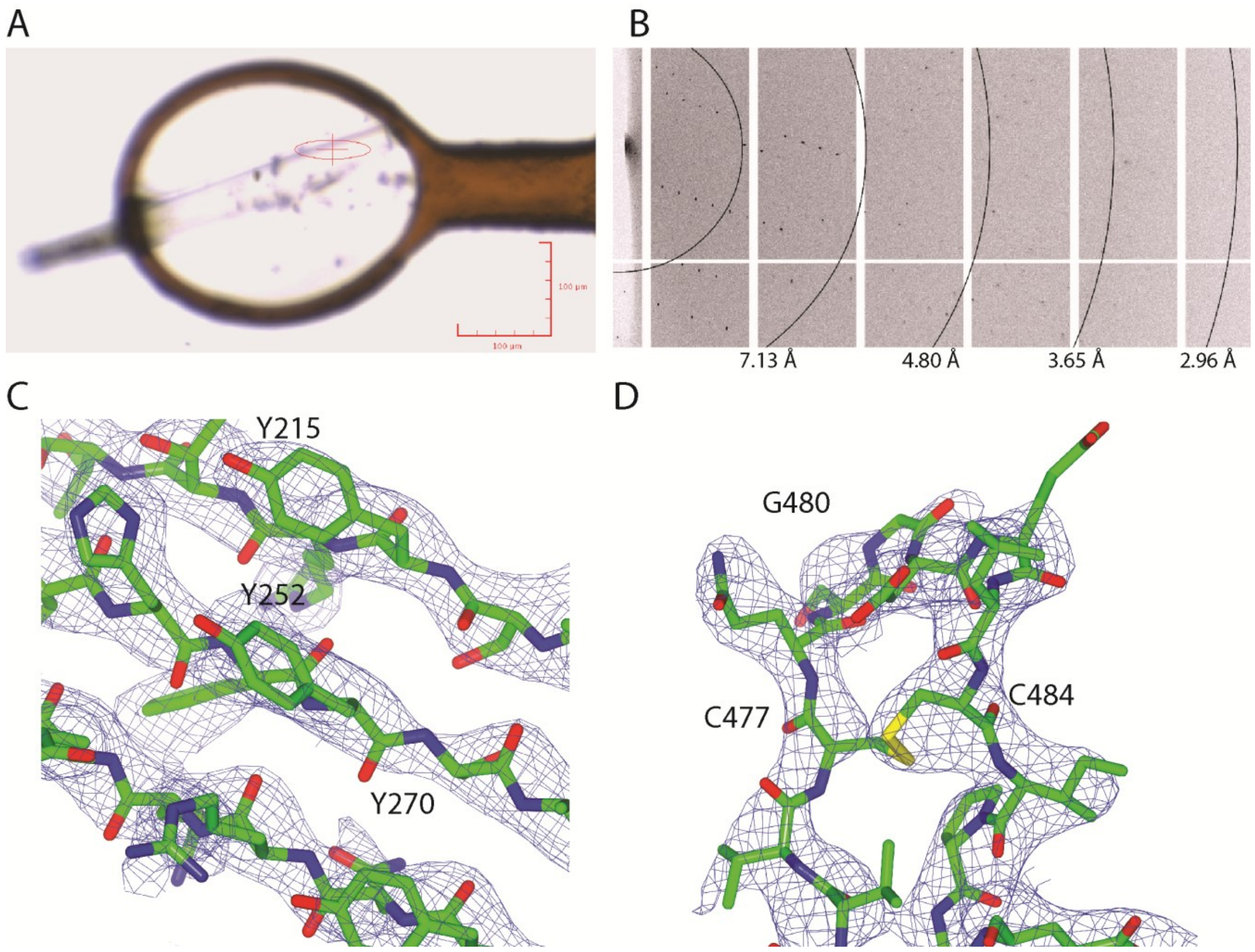

Supplementary Figure 4: FusA crystal structure was build and refined using native data from a crystal at 3.2 Å. A) A native FusA crystal looped and cryocooled at 100 K, visualised at DLS beamline I02. B) Diffraction data collected from crystal shown in A), diffraction extended to around 3.0 Å, dataset processed to 3.2 Å. C) A stick/ribbon model of FusA and chicken wire electron density representation, showing a section of strands 1-3 of the FusA β-barrel, undulating electron density is observed for β-strands and clear density is present for the larger sidechains. D) A stick/ribbon model and chicken wire electron density representation, showing the end of extracellular loop 4, electron density is present for the main chain as well as for the disulphide bond formed by cystines 477 and 484. The electron density map in C and D is a simulated annealing omit map and is contoured to 1.2 σ.

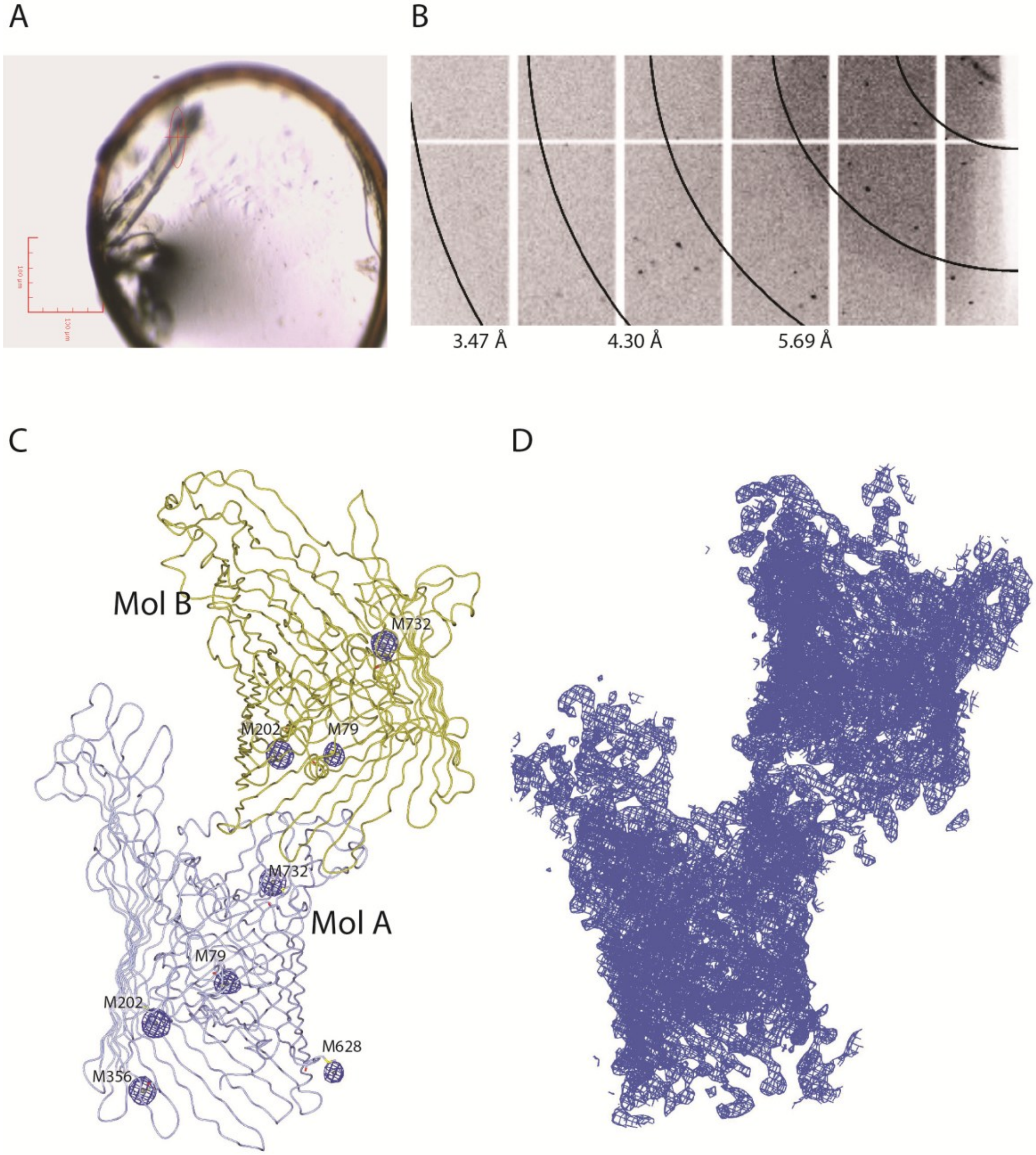

Supplementary Figure 5: FusA crystal structure phases obtained with SAD data from a K_2PtCl_4 soaked crystal at 4.27 Å. A) A FusA crystal soaked with K_2PtCl_4 looped and cryocooled at 100 K, visualised at DLS beamline I02. B) Diffraction data collected from crystal shown in A), diffraction extended to around 4 Å, dataset processed to 4.27 Å. C) Anomalous difference map derived from K_2PtCl_4 soaked dataset, 8 peaks are present in the map corresponding to 8 platinum atoms. All platinum atoms correspond to methionine residues from FusA, 5 from molecule A and 3 from molecule B of the crystallographic ASU, suggesting the platinum atoms have formed

covalent linkages to exposed methionines. D) Initial FusA density map from SAD phasing and density modification, clear density is observed from both the transmembrane β-barrel and extracellular loops.

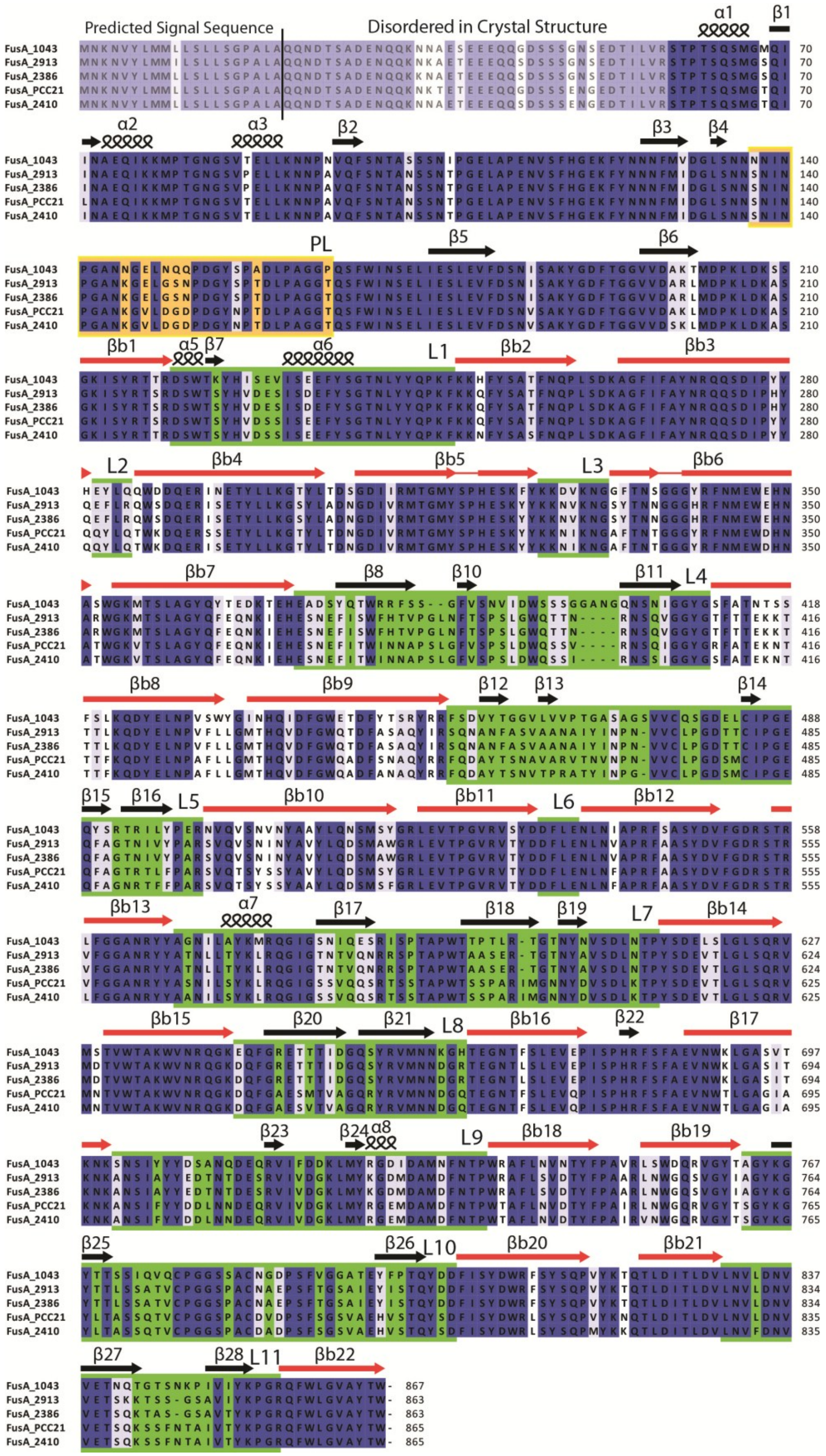

Predicted Signal Sequence
Disordered in Crystal Structure

Supplementary Figure 6: Sequence alignment of FusA homologues from different Pectobacterium strain shows a high level of sequence variation in its outer-loops. Sequence alignment of FusA homologues from different Pectobacterium isolates (FusA_1043 = FusA from P. atrosepticum SCRI 1043, CAG73790.1; FusA_2913 = FusA from *P. atrosepticum* LMG2913; FusA_2386 from *P. atrosepticum* LMG2386, KX258448; FusA_PCC21 = FusA from *P. carotovorum* PCC21, AFR02185.1; FusA_2410 = FusA from *P. carotovorum* LMG2410, KX258449) showing the location of secondary structure elements from the FusA crystal structure (arrow for β-sheets, loops for α-helices), β-sheets which form the FusA barrel are coloured in red. Extracellular loops are outlined in green and labelled L1 to L11, the plug domain loop is outlined in yellow labelled PL. The outer loops represent the regions of greatest sequence divergence between homologues.

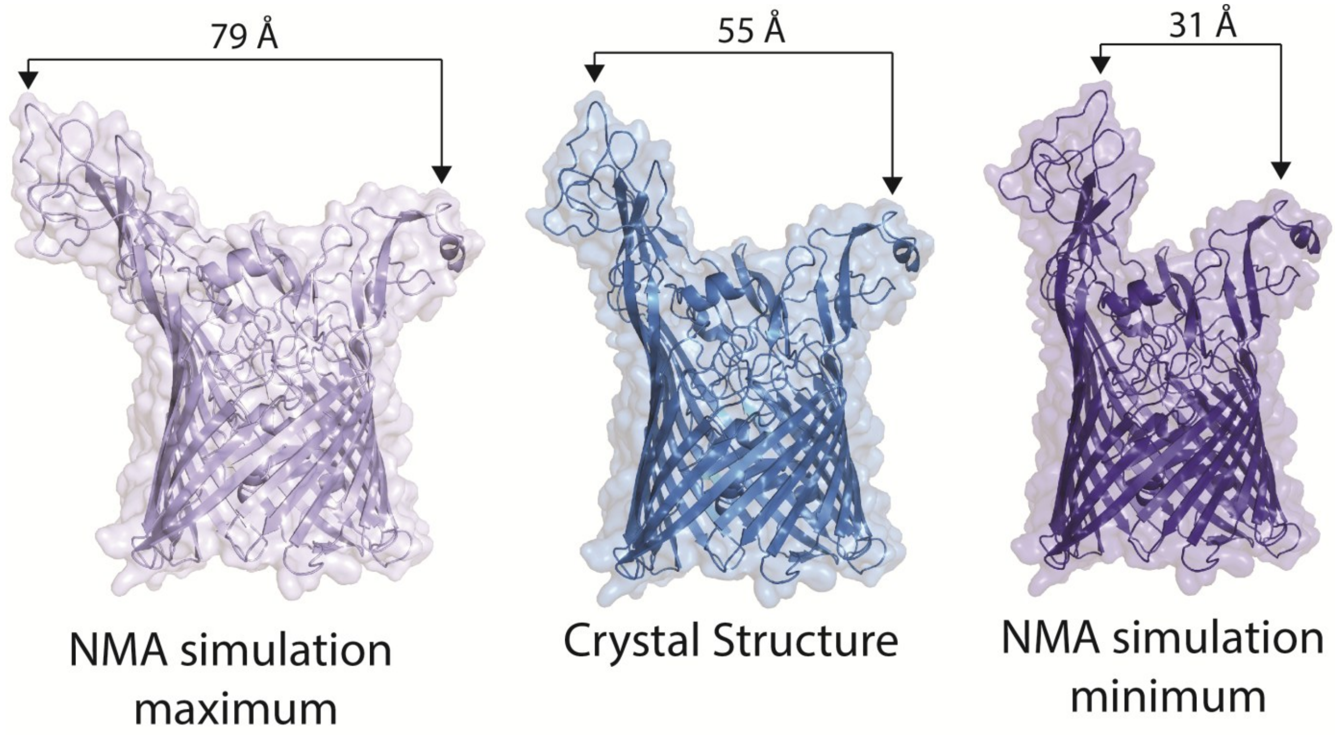

Supplementary Figure 7: Normal Mode Analysis of the FusA crystal structure identifies an opening and closing motion of the extracellular structures formed by loops 4,5 and 7, and loops 8-11. A cartoon/surface representation of the crystal structure of FusA at maximum and minimum extends of the NMA simulation, along with the unmodified crystal structure. The variation in distance of 79-31 Å between loops 5 and 10 on opposite sides of the β-barrel is indicative of the conformational change that FusA undergoes in the simulation.

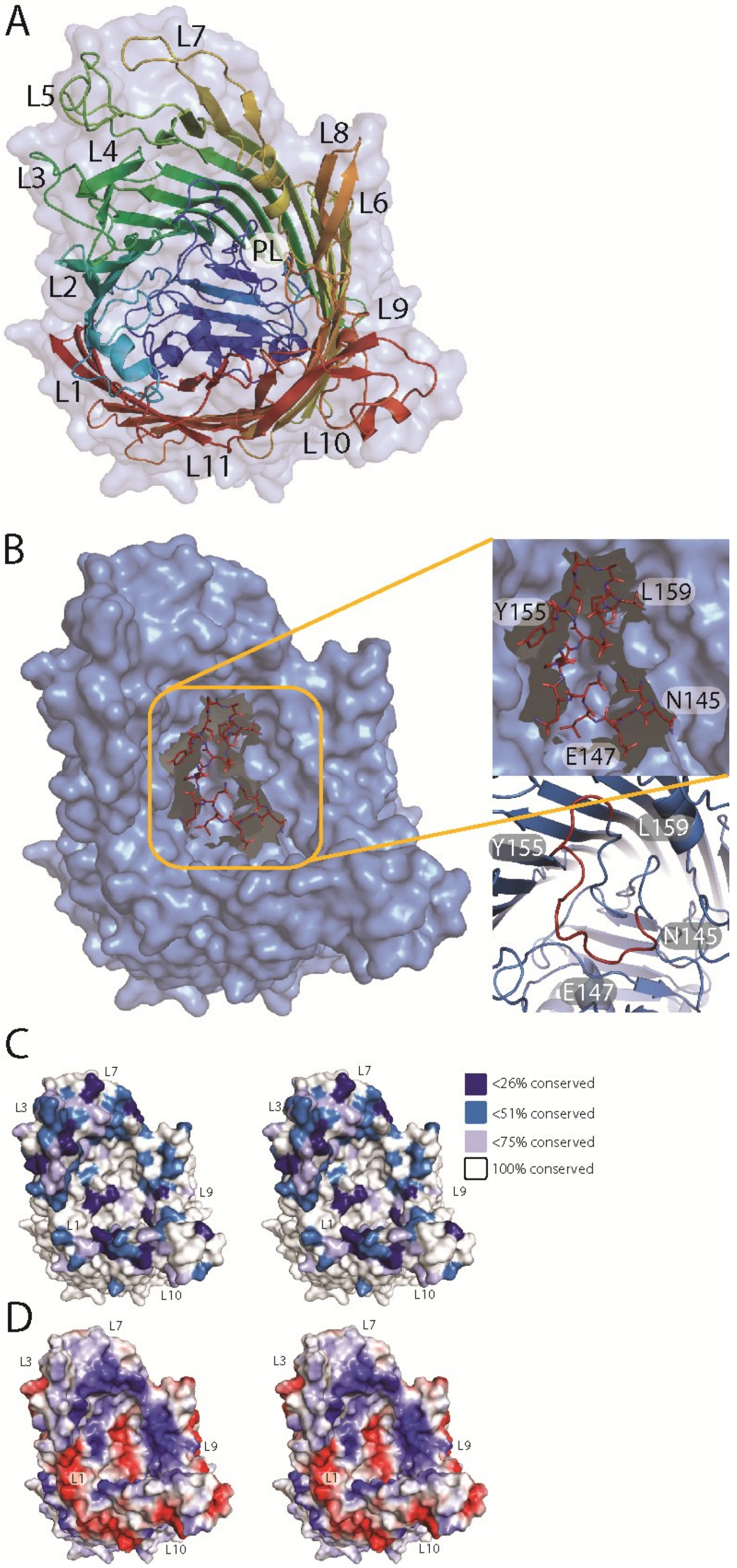
A
L7
L5
L8
L4
L3
L6
PL
L2
L9
L1
L10
L11
B
L159
Y155
N145
E147
L159
Y155
N145
E147
C
L7
L3
L9
L1
L10
<26% conserved
<51% conserved
<75% conserved
100% conserved
D
L7
L3
L9
L1
L10

Supplementary Figure 8: The outer membrane loops of FusA form a glove-like ferredoxin binding pocket, with an extended plug domain loop. A) Cartoon representation of extracellular region of FusA, with rainbow colouring showing N-terminus (blue) through C-terminus (red) with overlayed surface representation of FusA. Extraceullar loops are labelled L1-L11 and plug domain loop is labelled PL. B) Surface representation of FusA in the same orientation as A) showing the environmentally exposed residues of the plug domain as red sticks. A zoomed view of exposed plug loop residues is also shown in both stick/surface view and in cartoon representation. C) Stereo view of the surface of FusA in the same orientation as A), with extracellular loop residues which vary between FusA homologues in Supplementary Figure 2 mapped as: <26% conserved = dark blue, <51% conserved = sky blue, <76% conserved = light blue, conserved residues are shown as white. D) Stereo view of the surface of FusA in the same orientation as A showing the charged surface of the ferredoxin binding pocket, areas predicted to be negatively charged are show in red, while those predicted to be positively charged are shown in blue. The positively charged surface on the internal face of the pocket, is complementary to the predominantly negatively charged ferredoxin molecule.

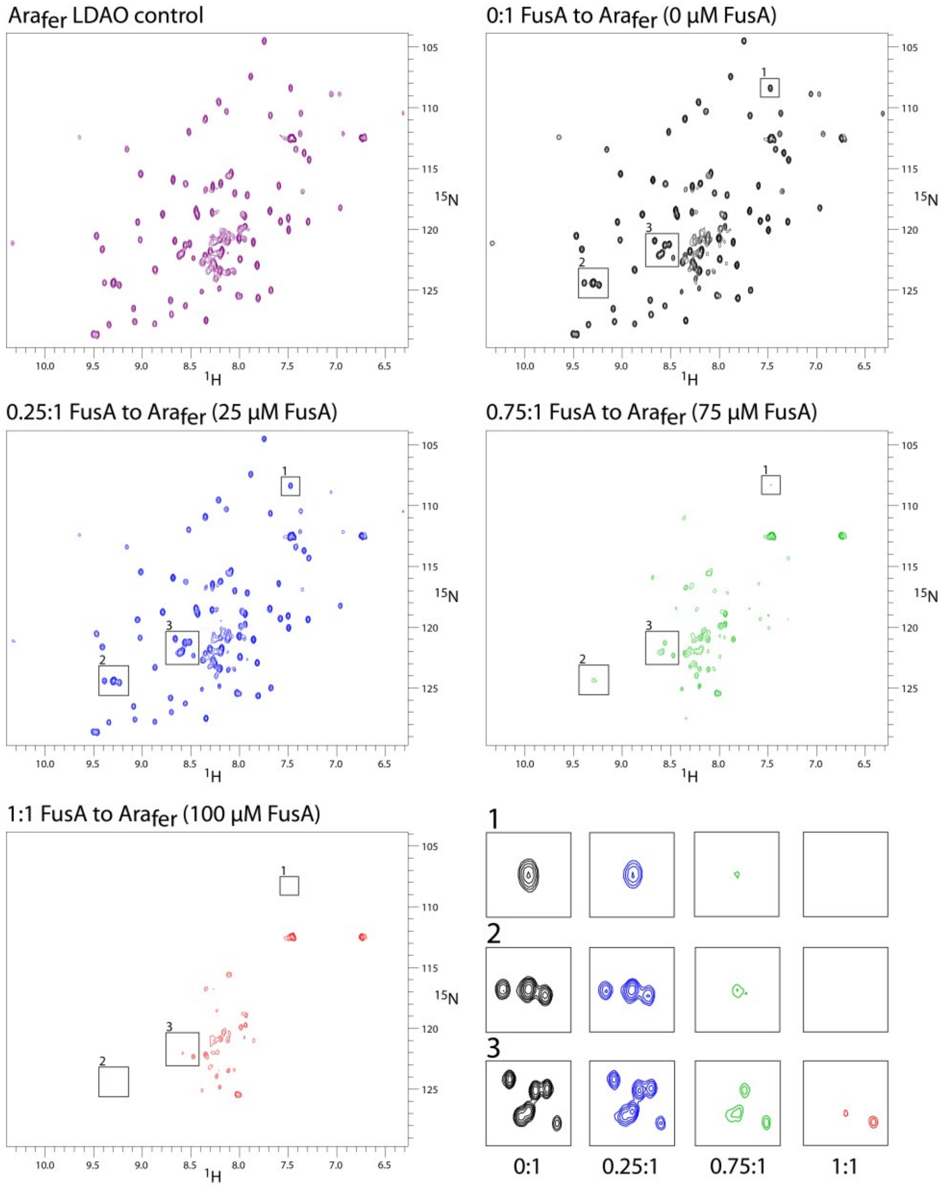

Supplementary Figure 9: NMR HSQC spectral analysis shows that Arabidopsis ferredoxin interacts with FusA at a 1:1 stoichiometry in a slow to intermediate exchange regime. The addition of FusA to ^{15}N labelled Fer$_{ara}$ lead to the broadening and intensity loss of peaks on the HSQC spectra in a concentration dependent fashion. Near complete loss of the Fer$_{ara}$ HSQC spectra at a Fer$_{ara}$ to FusA ratio of 1:1 suggests that binding occurs in a 1:1 stoichiometry. Due to detergent FusA interactions, increasing FusA concentration leads to an increased detergent (LDAO) concentration, as such a control was performed with Fer$_{ara}$ in buffer containing free LDAO micelles at a concentration comparable to that at a 0.75:1 ratio of FusA:Fer$_{ara}$, the intensity of HSQC spectra were not diminished significantly in the presence of the free LDAO micelles. (Experiment performed 3 times)

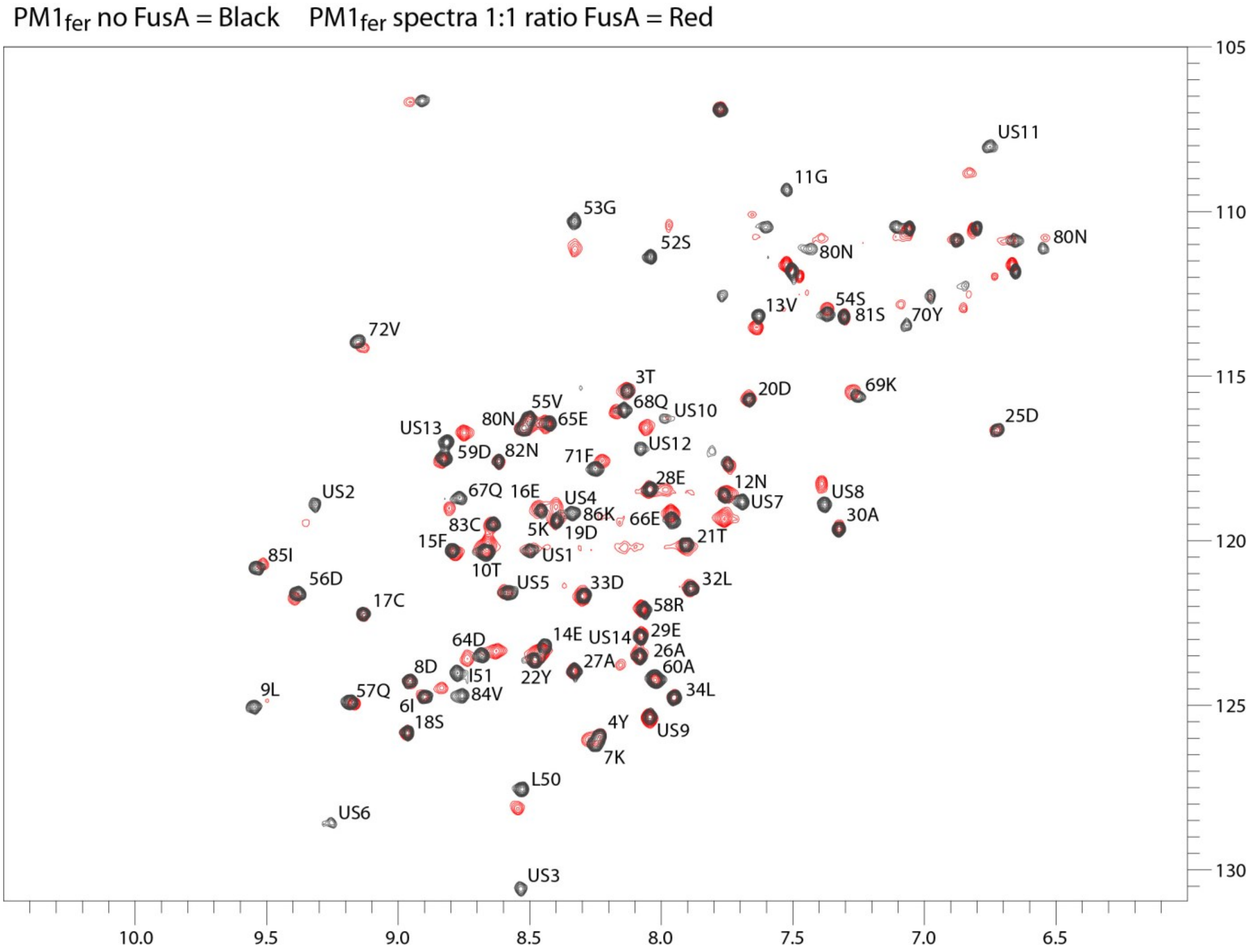

Supplementary Figure 10: Assigned NMR HSQC analysis shows that pectocin M1 ferredoxin domain interacts with FusA in a fast exchange regime, with chemical shifts observed for a subset of HSQC spectra peaks. The HSQC spectra of ^{15}N labelled $PM1_{fer}$ in the presence (red) and absence (black) of a 1:1 ratio molar ratio of FusA. The addition of FusA to ^{15}N labelled $PM1_{fer}$ leads to discrete chemical shifts and intensity loss in a subset of peaks on the $PM1_{fer}$ spectra, which is characteristic of binding between FusA and $PM1_{fer}$ in a fast exchange regime, suggestive of a binding affinity in the high µM to mM range. Residues corresponding to backbone resonances on the HSQC spectra were assigned, allowing for the identification of the FusA binding surface on $PM1_{fer}$. (Experiment performed 3 times).

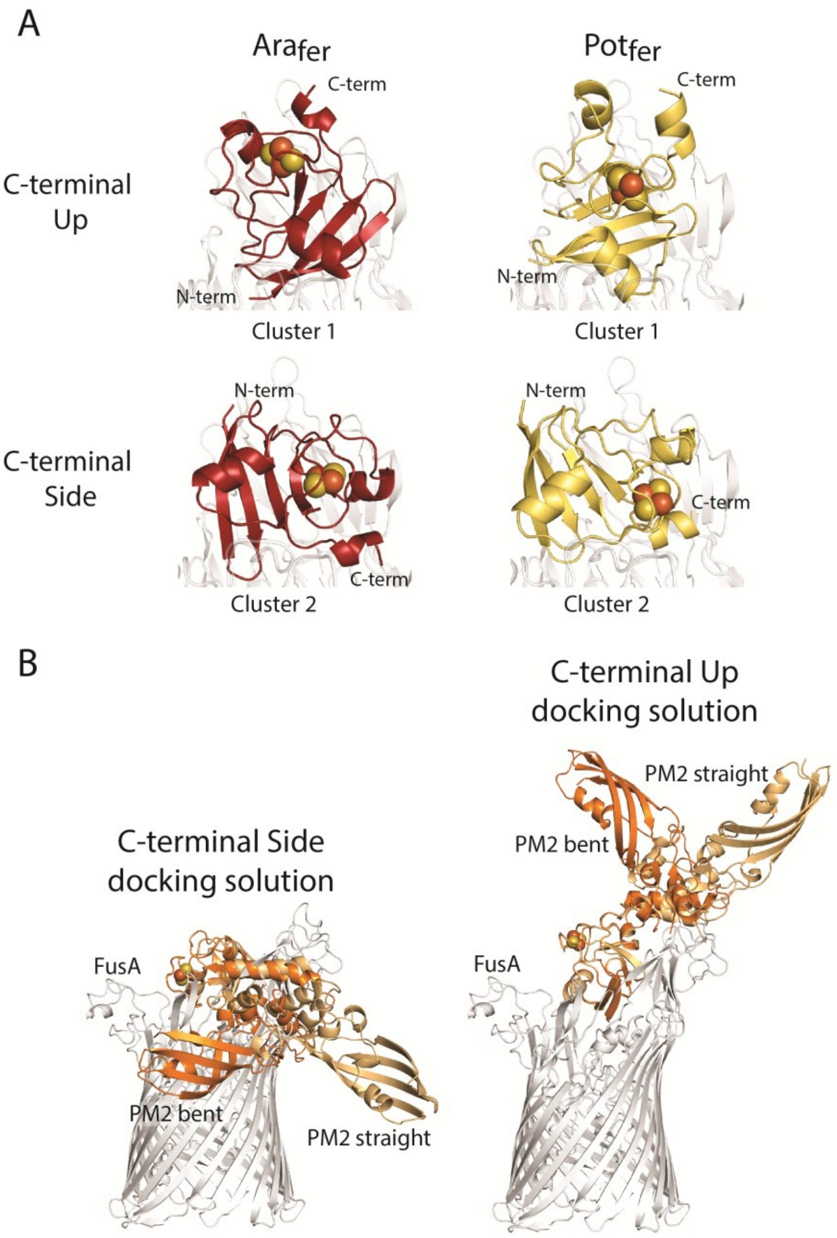

Supplementary Figure 11: CSP driven HADDOCK docking of ferredoxin to FusA provides a distinct solution independent of ferredoxin type. A) For ferredoxins from Arabidopsis and potato (Fer_{ara} and Fer_{pot}) utilised for FusA-ferredoxin using HADDOCK, two distinct solution clusters were obtained. In one the C-terminus of the ferredoxin points up and away from the FusA barrel and in the second it points sideways towards the side of the barrel. The first with the ferredoxin C-terminal pointing up was favoured for both ferredoxins (See Supplementary Table 2 for docking statistics). B) Superimposition of the crystal structures of pectocin M2 (PM2) in bent and elongated conformations onto HADDOCK docked ferredoxins. The crystal structure of pectocin M2 in both conformations is best accommodated in the C-terminal up solution.

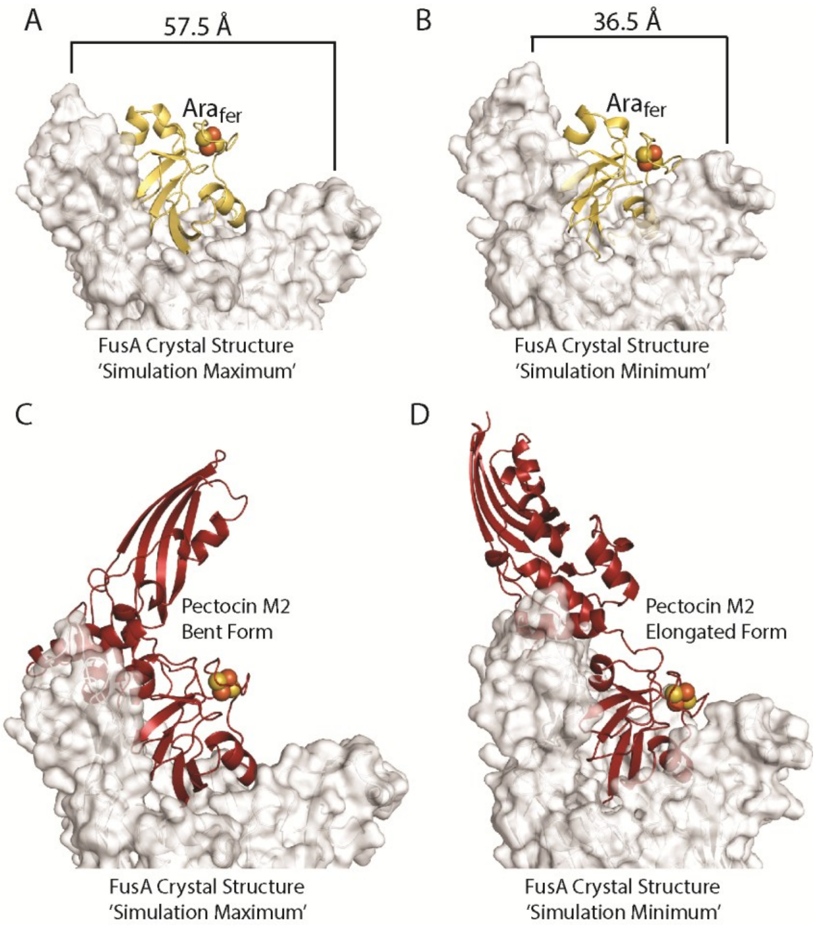

Supplementary Figure 12: Normal mode analysis of docked FusA and ferredoxin complex shows FusA encloses ferredoxin with its outer loops. A) Fer$_{ara}$ docked into the crystal structure of FusA forms extensive interactions with the β-wall structure formed by loops 4, 5 and 7, but limited interactions with the rest of the protein. B) When the docked structure is subjected to normal mode analysis the FusA glove closes around the ferredoxin domain forming extensive contacts with the remainder of Fer$_{ara}$. C) The bent form of pectocin M2 superimposed with docked PM1$_{fer}$ is accommodated well by the open FusA glove, while D) the closed glove conformation accommodates the elongated form of pectocin M2, suggesting that the flexibility of pectocin M2 is important for initial binding to FusA.

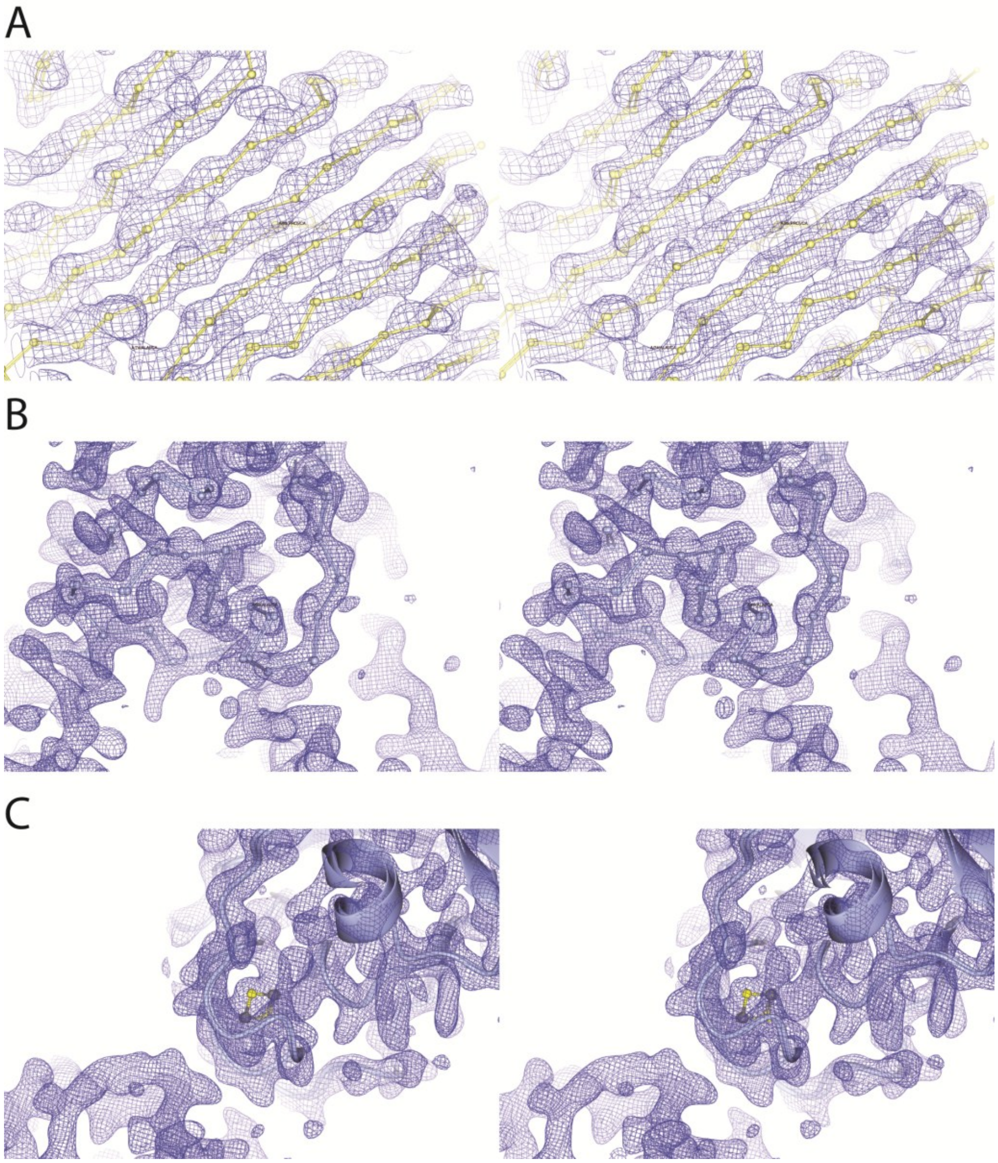

Supplementary Figure 13: Representative electron density from presented structures. A) Stereo view of 2Fo-2Fc electron density map from FusA crystal at 3.2 Angstroms resolution. Density contoured to 1.5 sigma. B) Stereo view of 2Fo-2Fc electron density map from Arabidopsis ferredoxin crystal at 2.34 Angstroms resolution. Density contoured to 1.5 sigma. C) Stereo view of 2Fo-2Fc electron density map from Potato ferredoxin crystal at 2.46 Angstroms resolution. Density contoured to 1.5 sigma.

Supplementary Table 1: A representative subset of FusA homologues identified based on sequence identity. FusA homologues identified by HMMER search of the Uniprot rp75 database, homologues are scored for the presence of the predicted periplasmic protease FusC adjacent to FusA in the genome. The arrangement of the putative Fus operon for these homologues is also noted in the 'Genomic context' column. The CLANS cluster to which the homologue localised (Shown in Supplementary Figure 2) is shown.

ID	Organism	Length	FusC Present	Genomic Context	E-value	% Identity to FusA	CLANS Cluster #
Q6D8U4_PECAS	Pectobacterium atrosepticum SCRI1043	860	Yes	*fusA/fusC/fusD*	0.00E+00	100	1
A0A0G4JQM7_9ENTR	Brenneria goodwinii	863	Yes	*fusA/fusC/fusD*	0.00E+00	65	1
A0A0Q4NF25_9ENTR	Erwinia sp. Leaf53	851	Yes	*fusA/fusC/fusD*	0.00E+00	61	1
E0SB25_DICD3	Dickeya dadantii (strain 3937)	856	Yes	*fusA/fusC/fusD*	0.00E+00	61.5	1
E6WMM1_PANSA	Pantoea sp. (strain At-9b)	856	Yes	*fusA/fusC/fusD*	0.00E+00	58.1	1
A0A085IFT4_9ENTR	Kluyvera ascorbata ATCC 33433	850	Yes	*fusA/fusC/fusD*	0.00E+00	56	1
A0A085JGB4_9ENTR	Tatumella ptyseos ATCC 33301	847	Yes	*fusA/fusC*	0.00E+00	56.3	1
I3DAK0_9PAST	Pasteurella bettyae CCUG 2042	826	Yes	*fusA/fusC*	3.40E-221	39.6	1
F9Q9W7_9PAST	Haemophilus pittmaniae HK 85	834	Yes	*fusA/fusC/fusD*	3.10E-220	40	1
W0Q4C4_9PAST	Mannheimia varigena USDA-ARS-USMARC-1261	834	Yes	*fusA/fusC/fusD*	6.10E-219	39.2	1
D0KIJ2_PECWW	Pectobacterium wasabiae WPP163	935	Yes	*fusA/fusC/fusD*	3.80E-217	37.4	1
A0A0B2T5W8_PECCA	Pectobacterium carotovorum	929	Yes	*fusA/fusC/fusD*	3.50E-216	37.4	1
A0A0A3AU62_9PAST	Chelonobacter oris	830	Yes	*fusA/fusC*	2.30E-211	39.7	1
A0A0T9TRQ5_YERFR	Yersinia frederiksenii	932	Yes	*fusA/fusC/fusD*	1.40E-210	37.5	1
A0A0T7NXT2_YEREN	Yersinia enterocolitica	932	Yes	*fusA/fusC/fusD*	2.20E-210	37	1
A6VQE6_ACTSZ	Actinobacillus succinogenes 130Z	843	Yes	*fusA/fusC*	2.20E-196	36.6	1
A0A0A7MF20_ACTEU	Actinobacillus equuli subsp. equuli	836	Yes	*fusA/fusC/fusD*	2.00E-195	37.4	1
M4R439_BIBTR	Bibersteinia trehalosi USDA-ARS-USMARC-192	855	Yes	*fusA/fusC/fusD*	3.10E-171	35.1	1
G4CPX8_9NEIS	Neisseria wadsworthii 9715	837	No	-	9.50E-169	31.7	1
A0A0N8IEC7_9NEIS	Neisseria sp. 74A18	837	No	-	2.70E-168	31.1	1
E8KEJ1_9PAST	Actinobacillus ureae ATCC 25976	877	Yes	*fusD/fusA/fusC*	1.90E-156	30.6	1
A0A011MII2_MANHA	Mannheimia haemolytica serotype A1/A6 str. PKL10	830	No	-	7.80E-153	29.9	1
Q7VNB0_HAEDU	Haemophilus ducreyi 35000HP	831	No	-	4.00E-143	28.6	1
M4RBG9_BIBTR	Bibersteinia trehalosi USDA-ARS-USMARC-192	894	No	-	2.60E-142	30.5	1
D4H693_DENA2	Denitrovibrio acetiphilus DSM 12809	845	No	-	2.90E-114	24.1	1b
A0A0C2DSN3_9DELT	Geoalkalibacter ferrihydriticus DSM 17813	839	No	-	3.90E-110	23.6	1b
W0H8S1_PSECI	Pseudomonas cichorii JBC1	944	Yes	*fusA/fusC/fusD*	1.10E-105	21.2	2
A0A077LRF4_9PSED	Pseudomonas sp. StFLB209	942	Yes	*fusA/fusC/fusD*	1.80E-104	20.8	2

A7GZS5 CAMC5	Campylobacter curvus (strain 525.92)	862	Yes	*fusC/_/_/fusA*	4.30E-104	24.3	2
A0A081Y721 9PSED	Pseudomonas sp. AAC	942	Yes	*fusA/fusC/fusD*	6.70E-104	21.3	2
A0A0J6H2R5 9PSED	Pseudomonas lundensis	866	No	-	1.60E-103	22.1	2
A0A0D1LVZ7 PSEPU	Pseudomonas putida	931	No	-	3.50E-103	21.1	2
U7A257 9PSED	Pseudomonas sp. CMAA1215	905	No	-	1.20E-102	21.4	2
A0A0W0NJ75 9PSED	Pseudomonas sp. ABAC61	878	No	-	1.90E-102	21.6	2
A0A0J6GD05 9PSED	Pseudomonas deceptionensis	866	No	-	1.00E-101	22	2
Q4KAL9 PSEF5	Pseudomonas protegens Pf-5	931	No	-	2.60E-101	20.5	2
A0A0B3BX93 9PSED	Pseudomonas tuomuerensis	881	No	-	8.90E-100	22.6	2
A0A0D0LNK2 PSEVI	Pseudomonas viridiflava	943	Yes	*fusA/fusC/fusD*	9.00E-100	21.9	2
A0A0J6IHX5 9PSED	Pseudomonas helleri	866	No	-	3.50E-99	21.6	2
A0A0E9ZNU4 9PSED	Pseudomonas sp. 10-1B	877	Yes	*fusA/fusC/fusD*	8.50E-99	21.9	2
A0A071LQD8 9PROT	Campylobacter mucosalis	842	No	-	1.20E-98	23.4	2
A0A0N0VIM9 9PSED	Pseudomonas fuscovaginae	979	Yes	*fusA/fusC/fusD*	3.10E-98	21.5	2
A7GZT3 CAMC5	Campylobacter curvus (strain 525.92)	859	Yes	*fusA/_/_/_/_/fusC*	1.40E-96	23.2	2
L1LW54 PSEPU	Pseudomonas putida CSV86	942	Yes	*fusA/fusC/fusD*	7.30E-95	20.2	2
U3HAC1 PSEAC	Pseudomonas alcaligenes OT 69	928	Yes	*fusA/fusC/fusD*	8.40E-95	21.9	2
A0A031IRG2 9PSED	Pseudomonas sp. RIT357	939	Yes	*fusA/fusC/fusD*	1.10E-94	20.2	2
C8PKD5 9PROT	Campylobacter gracilis RM3268	908	Yes	*fusC/fusA*	1.80E-94	21.2	2
M5CMP4 STEMA	Stenotrophomonas maltophilia SKK35	929	No	-	3.20E-91	21.4	2
A0A0B0Q4K4 9PROT	Sulfurospirillum sp. MES	905	Yes	*fusA/fusC/fusD*	6.30E-91	22.5	1c
B8L6A7 9GAMM	Stenotrophomonas sp. SKA14	927	No	-	8.30E-91	21.3	2
Q3A369 PELCD	Pelobacter carbinolicus DSM 2380	859	No	-	9.70E-91	20.2	1b
D1B2C7 SULD5	Sulfurospirillum deleyianum DSM 6946	908	Yes	*fusA/fusC/fusD*	2.80E-90	20.6	1c
A0A0D0L3H7 9PSED	Pseudomonas fulva	890	Yes	*fusA/fusC/fusD*	1.10E-89	21.6	2
G8QFJ2 DECSP	Dechlorosoma suillum PS	948	No	-	4.30E-88	21.3	2
N6YXS6 9RHOO	Thauera linaloolentis 47Lol = DSM 12138	925	No	-	4.80E-84	19.6	2
A0A0X3THZ3 9GAMM	Thiomicrospira sp. XS5	842	No	-	4.90E-83	23.3	-
A0A071LLD9 CAMHY	Campylobacter hyointestinalis subsp. hyointestinalis	841	No	-	2.10E-80	21.3	2
A0A0T7NV82 YEREN	Yersinia enterocolitica	921	Yes	*fusA/fusD/fusC*	2.50E-80	22.3	3
C4TZ44 YERKR	Yersinia kristensenii ATCC 33638	923	Yes	*fusA/fusD/fusC*	1.10E-79	22.1	3
A0A108U5W4 9GAMM	Lysobacter capsici AZ78	842	No	-	3.80E-79	20.2	2
B7URM8 ECO27	Escherichia coli O127:H6 str. E2348/69	790	Yes	*fusD/fusA/fusC*	4.90E-79	22.7	4
A0A0H5M188 YERIN	Yersinia intermedia	923	Yes	*fusA/fusD/fusC*	6.60E-79	22	3
A0A0H2YZ92 ECOK1	Escherichia coli O1:K1 / APEC	790	Yes	*fusD/fusA/fusC*	6.80E-79	22.7	4
Q1RBS7 ECOUT	Escherichia coli UTI89	790	Yes	*fusD/fusA/fusC*	6.80E-79	22.7	4
A0A0H2V8M0 ECOL6	Escherichia coli CFT073	790	Yes	*fusD/fusA/fusC*	7.60E-79	22.7	4
B7MMX0 ECO45	Escherichia coli S88	790	Yes	*fusD/fusA/fusC*	8.00E-79	22.7	4
B9CZF6 CAMRE	Campylobacter rectus RM3267	856	No	-	1.10E-78	20.7	2
Q8XAV9 ECO57	Escherichia coli O157:H7	790	Yes	*fusD/fusA/fusC*	1.30E-78	22.6	4
H1BXM2 ECOLX	Escherichia coli 4 1 47FAA	790	Yes	*fusD/fusA/fusC*	2.00E-78	22.6	4
D7XG58 ECOLX	Escherichia coli MS 198-1	790	Yes	*fusD/fusA/fusC*	2.00E-78	22.6	4

I2UG94_ECOLX	Escherichia coli 4.0522	790	Yes	*fusD/fusA/fusC*	2.60E-78	22.5	4
I2WAA6_ECOLX	Escherichia coli 9.0111	790	Yes	*fusD/fusA/fusC*	3.00E-78	22.5	4
F4V204_ECOLX	Escherichia coli TA280	790	Yes	*fusD/fusA/fusC*	3.20E-78	22.5	4
W1F5Y2_ECOLX	Escherichia coli ISC7	790	Yes	*fusD/fusA/fusC*	3.60E-78	21.7	4
A0A0I5GT18_SHISO	Shigella sonnei	790	Yes	*fusD/fusA/fusC*	4.90E-78	22.5	4
I2RMM2_ECOLX	Escherichia coli 1.2741	790	Yes	*fusD/fusA/fusC*	6.20E-78	22.5	4
A0A0T9LBC9_9ENTR	Yersinia nurmii	921	Yes	*fusA/fusD/fusC*	1.10E-77	22.3	3
E1J951_ECOLX	Escherichia coli MS 124-1	790	Yes	*fusD/fusA/fusC*	2.20E-77	22.4	4
A1JIL1_YERE8	Yersinia enterocolitica subsp. enterocolitica 8081	924	Yes	*fusA/fusD/fusC*	3.00E-77	21.8	3
YDDB_ECOLI	Escherichia coli (strain K12)	790	Yes	*fusD/fusA/fusC*	7.90E-77	22.3	4
E7TEJ9_SHIFL	Shigella flexneri CDC 796-83	790	Yes	*fusD/fusA/fusC*	1.90E-76	22.1	4
B2U1N6_SHIB3	Shigella boydii CDC 3083-94	790	Yes	*fusD/fusA/fusC*	4.40E-76	22.1	4
E7SG21_SHIDY	Shigella dysenteriae CDC 74-1112	790	Yes	*fusD/fusA/fusC*	4.50E-76	22.1	4
I6BR57_SHIFL	Shigella flexneri 2850-71	790	Yes	*fusD/fusA/fusC*	4.80E-76	22.2	4
B7LRE9_ESCF3	Escherichia fergusonii ATCC 35469	790	Yes	*fusD/fusA/fusC*	8.10E-76	22.6	4
M4U9N6_9GAMM	Psychromonas sp. CNPT3	825	No	-	2.70E-75	21.6	3
I6DIA8_SHIBO	Shigella boydii 965-58	775	No	-	3.10E-75	22.1	4
Q83KV8_SHIFL	Shigella flexneri	786	No	-	9.10E-74	22	4
B4EXQ6_PROMH	Proteus mirabilis (strain HI4320)	916	Yes	*fusA/fusD/fusC*	2.20E-73	21.1	3
Q729G8_DESVH	Desulfovibrio vulgaris str. Hildenborough	875	No	-	1.20E-70	19.8	-
A0A0S2DGT0_LYSEN	Lysobacter enzymogenes	853	No	-	7.10E-70	19.4	2
I7GU01_9HELI	Helicobacter cinaedi CCUG 18818 = ATCC BAA-847	879	No	-	9.40E-70	19.2	2
A0A099U869_9HELI	Helicobacter magdeburgensis	881	No	-	3.60E-69	19.2	2
A6W1Y0_MARMS	Marinomonas sp. (strain MWYL1)	934	Yes	*fusA/fusC*	4.20E-69	17.9	-
T1D0M6_9HELI	Helicobacter fennelliae MRY12-0050	905	No	-	4.90E-69	19.6	2
K2JWF4_9GAMM	Gallaecimonas xiamenensis 3-C-1	873	No	-	2.10E-68	19	-
C0AZ86_9ENTR	Proteus penneri ATCC 35198	886	No	-	2.20E-68	21	3
D4C1N0_PRORE	Providencia rettgeri DSM 1131	921	Yes	*fusA/fusD/fusC*	2.30E-68	20.5	3
D4SQL7_9XANT	Xanthomonas fuscans subsp. aurantifolii str. ICPB 11122	849	No	-	3.90E-68	19	2
F5NVB3_SHIFL	Shigella flexneri K-227	739	Yes	*fusD/fusA/fusC*	4.60E-68	20	4
A0A099BER5_9HELI	Helicobacter sp. MIT 01-6451	888	No	-	4.60E-68	20.5	2
A0A099UEA1_9HELI	Helicobacter typhlonius	884	No	-	2.50E-67	20.7	2
D1P6G3_9ENTR	Providencia rustigianii DSM 4541	819	Yes	*fusA/fusD/fusC*	5.30E-67	21.5	3
A0A0Q4NS92_9BURK	Duganella sp. Leaf61	929	Yes	*fusA/fusC*	5.60E-67	18	2
A0A0H1AWM2_9GAMM	Luteimonas sp. FCS-9	864	No		9.30E-67	18.6	2
Q315Z8_DESAG	Desulfovibrio alaskensis (strain G20)	839	Yes	*fusC/_/_/fusA*	5.60E-66	19.7	-
K8W959_PRORE	Providencia rettgeri Dmel1	917	Yes	*fusA/fusD/fusC*	1.60E-65	20.6	3
Q3IJB3_PSEHT	Pseudoalteromonas haloplanktis TAC125	906	No	-	1.80E-65	18.2	-
A0A0L6X5Z0_PROST	Providencia stuartii	922	Yes	*fusA/fusD/fusC*	1.80E-65	20.3	3

J7TNK4_MORMO	Morganella morganii subsp. morganii KT	928	Yes	*fusA/fusD/fusC*	2.90E-65	20.3	3
A0A0Q0GJ23_9GAMM	Shewanella sp. P1-14-1	920	Yes	*fusA/fusC*	6.10E-65	18.1	-
K8W1D2_9ENTR	Providencia sneebia DSM 19967	921	Yes	*fusA/fusD/fusC*	8.30E-65	20.4	3
W3YHJ0_9ENTR	Providencia alcalifaciens PAL-3	917	Yes	*fusA/fusD/fusC*	1.60E-64	20.8	3
K8W4G5_9ENTR	Providencia burhodogranariea DSM 19968	923	Yes	*fusA/fusD/fusC*	5.60E-64	20.2	3

Supplementary Table 2: HADDOCK driven ferredoxin to FusA docking statistics. PDB coordinates for a representative docking results for each cluster is provided as a supplementary dataset.

**Ferredoxin**	**Arabidopsis**		**Potato**	
Cluster number	1	4	1	2
Cluster population	151/180	7/180	123/189	44/189
C-terminal orientation	Up	Side	Up	Side
RMSD from overall lowest-energy structure (Å)	5.8 ± 0.1	1.4 ± 0.8	5.4 ± 0.3	1.1 ± 0.8
HADDOCK score (a.u.)	-137.8 ± 2.1	-130.8 ± 20.3	-148.5 ± 3.0	-145.7 ± 3.0
Van der Waals energy (kcal mol^{-1})	-72.5 ± 10.5	-70.4 ± 17.9	-75.9 ± 7.8	-91.6± 5.9
Electrostatic energy (kcal mol^{-1})	-476.2 ± 66.5	-494.9 ± 39.9	-504.3 ± 55.5	-387.2 ± 50.3
Desolvation energy (kcal mol^{-1})	28.9 ± 10.0	33.9 ± 14.7	26.4 ± 13.0	21.3 ± 13.0
Restraints violation energy (kcal mol^{-1})	11.2 ± 9.62	47.2 ± 29.71	18.0 ± 10.98	19.6 ± 11.93
Buried surface area ($Å^2$)	2524.8 ± 175.9	2728.9 ± 345.5	2694.9 ± 190.4	2882.4 ± 156.6

Supplementary Table 3: Growth enhancement of Pectobacterium strains by ferredoxins originating from Arabidopsis, Maize and Potato. The plant ferredoxins were tested against a panel of Pectobacterium isolates for their ability to enhance growth under iron limiting conditions . Serial dilutions of purified ferredoxin was spotted on LB agar, depleted of free iron by the addition of 200 µM Bipryidine, seeded with a lawn of Pectobacterium cells. The ferredoxins were scored for the 'Strength' of the effect (The relative strength of the zone of growth enhancement compared to the most pronounced zone (that of LMG2410)) and the lowest dilution at which growth enhancement was observed. (Experiment performed >10 times, data from 1 representative experiment).

				**Arabidopsis**		**Potato**		**Maize**	
**Species**	**Host of Isolation**	**Collection**	**Strain #**	**Dilution (mg/ml)**	**Strength**	**Dilution (mg/ml)**	**Strength**	**Dilution (mg/ml)**	**Strength**
P. atrosepticum	Potato	SCRI	1043	0.04	Weak	0.33	Weak		
P. atrosepticum	Celery	LMG	2374						
P. atrosepticum	Potato	LMG	2378	0.33	Weak				
P. atrosepticum	Potato	LMG	2383						
P. atrosepticum	Potato	LMG	2384	0.04	Medium	0.11	Weak		
P. atrosepticum	Potato	LMG	2385	0.04	Weak	0.33	V Weak		
P. atrosepticum	Potato	LMG	2386	0.04	Weak				
P. atrosepticum	Soil	LMG	2390	0.11	Weak				
P. atrosepticum	Soil	LMG	2391	0.33	Weak				
P. carotovorum	Cucumber	LMG	2410	0.04	Strong	0.04	Medium		
P. carotovorum	Hyacinth	LMG	2412	0.04	Strong	0.04	Medium		
P. carotovorum	Cabbage	LMG	2442	0.04	Strong	0.04	Strong	0.33	Weak
P. carotovorum	Cabbage	LMG	2443	0.04	Strong	0.04	Weak		
P. carotovorum	Potato	LMG	2444	0.04	Weak	1.00	Weak		
P. carotovorum	Chrysanthemum	LMG	2454	0.33	Weak				
P. carotovorum	Soil	LMG	2913	0.04	Medium	0.11	Weak		
Erwinia rhapontici	Rhubarb	LMG	2686						
